# Comparative Evaluation of Shade Selection Reliability: Visual Assessment, Intraoral Scanning, and Photographic Techniques

**DOI:** 10.1155/ijod/5516548

**Published:** 2026-05-12

**Authors:** Faezeh Atri, Faraz Rahmani, Maryam Memarian, Parsa Pirooz

**Affiliations:** ^1^ Department of Prosthodontics, School of Dentistry, Tehran University of Medical Sciences, Tehran, Iran, tums.ac.ir; ^2^ Craniomaxillofacial Research Center, Shariati Hospital, Tehran University of Medical Sciences, Tehran, Iran, tums.ac.ir; ^3^ Department of Endodontics, School of Dentistry, Guilan University of Medical Sciences, Rasht, Iran, gums.ac.ir

**Keywords:** color, dental, esthetics, photography

## Abstract

**Purpose:**

The critical role of color matching in prosthetic dentistry underscores the necessity of identifying the most dependable shade selection technique, thereby reducing the incidence of dissatisfaction in esthetic dental outcomes. This research evaluated the reliability of the field’s three predominant shade selection techniques (visual assessment, intraoral scanning, and photography).

**Materials and Methods:**

A group of 26 individuals with nearly pristine maxillary right central incisors (free from wear, discoloration, or enamel defects) aged between 20 and 40 years was selected for this study. The color matching of these teeth was conducted using three methods: visual assessment by three specialized evaluators, intraoral scanning, and photographic documentation (utilizing both a single‐lens reflex (SLR) camera and a smartphone). These procedures were repeated after a fortnight, and the reliability of each method was assessed and compared through the interclass correlation coefficient (ICC) test (with a significance threshold set at 0.05).

**Results:**

The outcomes revealed a notable disparity in the reliability of different shade selection approaches (*p*  < 0.05). Reliability ratings were as follows: intraoral scanning at 92.3%, visual assessment at 61.5%, SLR camera photography at 15.4%, and smartphone photography at 7.7%. Although the results initially favored visual assessment, this method suffered significant inter‐evaluator reliability issues.

**Conclusions:**

The findings suggest that intraoral scanning is the most reliable method for accurate color determination of teeth, outperforming visual assessments. The use of photographic images, regardless of the capture device, proved to be the least reliable method.

**Clinical Significance:**

Achieving an exact color match is crucial for the success of any restorative dental procedure. The most reliable method for this precision is through an intraoral scanner.

## 1. Introduction

Replicating the natural hue of teeth remains a significant challenge for clinicians and dental technicians alike [1]. The demand for esthetic dental restorations has surged in recent years, driven by increased patient awareness and significant advancements in restoration materials and techniques, allowing for more esthetically pleasing outcomes [2]. Incorrect shade selection is a prevalent failure cause [3–5], and it is reported as the second most common cause of replacing ceramic dental restorations [6, 7].

The traditional approach to shade selection involves commercial shade guides, a method susceptible to subjectivity, observer fatigue, the influence of ambient lighting, and the metamerism phenomenon [7]. The VITA Classical shade guide (VITA Zahnfabrik, Germany) has been widely utilized, covering a broad spectrum of materials used in ceramic and composite restorations [8]. In 1998, to enhance color matching capabilities and include a broader spectrum of colors, the VITA 3D Master Shade Guide (VITA Zahnfabrik, Germany) was introduced, providing a more systematic approach to shade selection. However, both guides are suitable for prosthetic dental needs [8].

Alternative methods for shade selection, such as photography with single‐lens reflex (SLR) cameras and using instruments like spectrophotometers, spectrocolorimeters, and spectroradiometers, offer an objective approach that could improve the precision and reliability of shade determination [9]. Despite being highlighted as the most accurate and precise method among instrumental and visual approaches [10], spectrophotometric shade selection faces limitations in accessibility within dental clinics and communication between the clinician and technician [11, 12]. These instruments are criticized for their uneven distribution and insufficient dental shade spectrum coverage [13].

Digital camera photography has become widely accepted among clinicians to facilitate communication regarding teeth morphology, color distribution on the teeth surface, and intraoral conditions between clinicians and dental technicians [14].

The adoption of smartphones has markedly increased, driven by their various applications, wireless connectivity, high‐resolution photography capabilities, and ease of communication [15, 16]. However, it is noteworthy that for camera and smartphone photography, lighting circumstances should be controlled to make results reliable [17]. In parallel, intraoral scanners are becoming increasingly popular in dental practices, with some models capable of identifying tooth color and distinguishing between tooth structure and the oral cavity, potentially replacing traditional dental impressions [18].

While previous studies have compared shade selection methods individually (e.g., visual technique vs. spectrophotometry [19], spectrophotometry vs. SLR camera [6], visual technique vs. intraoral scanner [20]), there has been a lack of clinical research that simultaneously includes visual assessment, intraoral scanning, SLR camera photography, and smartphone camera photography. Hence, this study aims to evaluate the reliability of these methods to determine the most effective one by comparing the obtained results. The null hypothesis proposed that no difference in reliability among the mentioned methods exists.

## 2. Methods and Materials

This research received approval from the Ethics Committee of Tehran University of Medical Sciences (ID: 9311272021) in 2022. The study participants were selected from a pool of volunteers from whom informed consent was obtained. Personal information was kept confidential to ensure privacy, and faces remained unidentifiable in photographs.

Twenty‐six individuals (14 female, 12 male, aged 20–40 years) with intact right central incisors were recruited for the study. The sample size was established based on a study by Liberato et al. [21] and calculated using interclass correlation power analysis (considering *α* = 0.05, *β* = 0.02, *R*_0_ = 0.3, *R*_1_ = 0.67). This yielded a minimum sample of 26 participants. The teeth selected for the study showed no signs of abrasion, pigmentation, or enamel anomalies. Shade selection focused on the middle third of the clinical crown of the teeth, both in the incisogingival and mesiodistal dimensions (the central one‐ninth of the teeth). All participants were in their twenties or thirties. Before examination, participants were instructed to brush their teeth and avoid wearing makeup. A comprehensive tooth polishing was also performed.

The evaluation employed three distinct methods: visual evaluation, intraoral scanning, and photography (utilizing both a smartphone and an SLR camera), each repeated after 2 weeks to ascertain reliability. Color properties were converted into CIELAB coordinates, reported as Lab values [22].

### 2.1. Visual Assessment Method

This evaluation was conducted by three clinicians, each with over 10 years of experience and who had passed the Ishihara color blindness test [12]. The evaluators were blinded to each other’s decisions, ensuring an unbiased process under consistent conditions: daylight lighting, a 45° angle of the dental chair, and a distance of 30 cm between patient and clinician. Shade selection was based on the VITA Classical shade guide A1–D4, prioritizing hue, chroma, and value. The Lab values for the chosen color were derived from the VITA Classical color guide table [10, 23].

### 2.2. Intraoral Scanning Method

The 3shape TRIOS A/S intraoral scanner (3Shape, Denmark) was calibrated using the TRIOS color calibration kit (3Shape, Denmark). The VITA Classical shade selection mode was activated in the scanner’s software (TRIOS 3). After scanning the right central incisor, the identified color was recorded, with Lab values determined using the VITA Classical color guide table [10, 23].

### 2.3. Photographic Shade Selection Method

Conducted separately using a Nikon D7100 SLR camera (Nikon, Japan) and a HUAWEI Y6 smartphone (Huawei, China). The SLR camera settings were standardized: 1:1 magnification ratio, automatic exposure mode, automatic white balance and aperture, TTL flash metering with manual flash mode off, high image resolution, JPEG file type with uniform image compression, and an ISO value of 125. The Coco Lux Mobile Dental Photography Light (Hass Bio, South Korea) provided daylight conditions for smartphone photography.

Both devices were positioned to capture images at a 30 cm distance, level with the occlusal plane, using a lip and cheek retractor to keep the patient’s mouth open and a standardized gray reference card (WhiBal, Michael Tapes Design) to cover the left central incisor and post hoc color calibration. The images were processed in Adobe Photoshop CC 2019, where Lab values were extracted following exposure calibration and white balancing with the gray reference card (*L* = 75, *a* = 0, *b* = 0) [24].

For all methods, the Lab values from the initial and follow‐up examinations were calculated using the Δ*E*_*ab*_ formula, and the Δ*E*_2000_ value was determined through an online calculator (http://colormine.org/delta-e-calculator/cie2000). The Δ*E*_2000_, an improved version of Δ*E*_*ab*_, correlates closely with human visual perception [25]. This index links measured color differences to the CIELAB coordinates [26]. The reliability of the shade selection methods was analyzed and compared using the interclass correlation coefficient (ICC) Test [27], with a significance level set at *p*  < 0.05. Additionally, the reliability of each examiner was compared.

## 3. Results

The Δ*E*_2000_ values were calculated for each participant across all methods, comparing the initial and subsequent shade selections over a 2‐week interval. Δ*E*_2000_ values can be interpreted according to the following criteria: values less than or equal to 0.8 indicate excellent color matching, values between 0.8 and 1.8 are considered acceptable, and values greater than 1.8 signify a mismatch. The mismatch category was subdivided into three ranges: mismatch type *a* = 1.8–3.6, mismatch type *b* = 3.6–5.4, and mismatch type *c* > 5.4 [28].

Table 1 displays the distribution of participants across each Δ*E*_2000_ category for every shade selection method. For the visual assessment method, results for each examiner were individually recorded, and the frequency of outcomes based on the examiners’ average values was provided in the final column.

**Table 1:** The frequency distribution of Δ*E*_2000_ categories for the shade selection methods.

Outcome	SLR camera	Smartphone	Scanner	Visual (examiner 1)	Visual (examiner 2)	Visual (examiner 3)	Visual (average)
Mismatch type c	8 (30.8%)	9 (34.6%)	0	1 (3.8%)	3 (11.3%)	0	0
Mismatch type b	10 (38.5%)	5 (19.2%)	0	8 (30.8%)	2 (7.7%)	4 (15.4%)	4 (15.4%)
Mismatch type a	4 (15.4%)	10 (38.5%)	2 (7.7%)	6 (23.1%)	7 (26.9%)	3 (11.5%)	6 (23.1%)
Acceptable match	4 (15.4%)	2 (7.7%)	2 (7.7%)	1 (3.8%)	0	2 (7.7%)	8 (30.8%)
Excellent match	0	0	22 (84.6%)	10 (38.5%)	14 (53.8%)	17 (65.4%)	8 (30.8%)
Excellent + acceptable match	4 (15.4%)	2 (7.7%)	24 (92.3%)	11 (42.3%)	14 (53.8%)	19 (73.1%)	16 (61.5%)

The Friedman test [29] was utilized for method comparison because the data did not conform to normal distribution (Table 2). A significant difference was observed among the shade selection methods evaluated (*p*‐value <0.05), indicating that the outcomes varied significantly across methods. Notably, the results from the scanner indicated no discrepancies.

**Table 2:** Friedman test results for the shade selection methods.

Shade selection method	Mean rank	Chi‐square	*p*‐Value
SLR camera	4.04	64.832	<0.001
Smartphone	4.04		
Scanner	1.29		
Visual (average)	2.19		

The ICC test results are summarized in Table 3. The ICC values with 95% confidence intervals arranged from lowest to highest are as follows: intraoral scanner, visual assessment, smartphone, SLR camera. With an ICC value significantly greater than zero (*p*‐value <0.05), there was an agreement between the different methods, suggesting that the study’s reliability is acceptable. An ICC value of 0.64 indicates moderate reliability when considering the average Δ*E*_2000_ values of the different methods. Among the shade selection methods, the SLR camera exhibited the highest mean Δ*E*_2000_ value (4.68), indicating less reliability in color matching.

**Table 3:** ICC test results for the shade selection methods.

Shade selection method	Mean	Standard deviation	ICC	CI 95%	*p*‐Value
SLR camera	4.68	2.18	0.64	0.365–0.82	<0.001
Smartphone	4.16	1.81			
Scanner	0.34	0.82			
Visual (average)	1.78	1.48			

Interpersonal reliability among the examiners using the visual assessment method was also evaluated using the ICC test, with the results presented in Table 4. With a *p*‐value of 0.023 (less than 0.05), there was an agreement among the examiners, though the ICC value (0.485) reflects the poor reliability of the visual assessment method overall. This suggests variability in the consistency of shade selection among different evaluators within the visual assessment method.

**Table 4:** ICC test results for the examiners of the visual shade selection method.

Examiner	Mean	Standard deviation	ICC	CI 95%	*p*‐Value
Examiner 1	2.26	2.06	0.485	0.012–0.752	0.023
Examiner 2	1.90	2.42			
Examiner 3	1.17	1.79			

## 4. Discussion

The null hypothesis of this study was rejected, as results identified the scanning method as the most reliable for shade selection, boasting a reliability score of 92.3%. The visual assessment method ranked second with 61.5% reliability, while the photographic shade selection methods via SLR cameras and smartphones lagged significantly behind, with 15.4% and 7.7% reliability, respectively.

Consistent with the findings of this study, several other investigations have corroborated the high reliability of scanner‐based shade selection (2, 18, and 27–29). Reyes et al. [2] compared visual shade selection with scanner‐based methods, reporting a scanner reliability of 86.66%, closely aligning with the 92.3% reliability found in the current study. Liberato et al. [21] also endorsed the scanner as a more reliable method than visual assessment, a conclusion that agrees with the findings. Their study, unlike ours, also explored the impact of using a light‐correcting device (Smile Lite), which was found to enhance the shade selection process. Similarly, studies by Czigola et al. [30] and Rutkūnas et al. [31] affirmed the trustworthiness of scanners for shade selection, with Rutkūnas et al. [31] reporting over 92% reliability, paralleling the study’s outcomes.

Besides the advantages of intraoral scanners, it is noteworthy that the accuracy of intraoral scanners is affected by some items, such as the scanner brand, operator’s skill, regular calibration, and patient mouth circumstance [32]. Variables like scanning duration, up‐to‐date software, scanning pace and strategy, surrounding lighting, and environment temperature, moreover, are important influential factors [32]. So, utilizing intraoral scanners for shade selection should be performed with exact control of confounding factors to bring to reliable outcome.

Brandt et al. [33] studied the accuracy of shade selection by visual assessment and scanner and took a spectrophotometer VITA Easy Shade (VITA Zahnfabrik, Germany) as the reference. This study was carried out on 107 central maxillary teeth. The shade selection accuracy was comparable across all methods and ranked from highest to lowest as follows: scanner, visual shade selection by the dentists, and visual shade selection by the technicians. It is worth noting that the accuracy of all these methods fell within the clinically acceptable range. Resulted accuracies are in agreement with the present study. In contrast with the current study, which calculated reliability for all of the methods, the reliability of only one method (scanning method) was determined by performing three rounds of shade selection on a segment of subjects (20 central teeth) (78.3%). The smaller subject group and an additional selection round might explain the lower reliability of the scanning method in comparison to our study.

While some studies have positioned photographic methods as superior to visual assessment, such conclusions conflict with current research findings [34, 35]. Jorquera et al. [34] found photography a more acceptable approach, a discrepancy that could be attributed to differences in the instruments used for photographs and the focus on healthy teeth. Kelkar et al. [35] compared visual assessment with digital and polarizing filter photography, concluding that photography yielded more accurate results. This divergence might stem from variations in examiner experience, camera types used, and the in vitro nature of Kelkar’s study compared to the clinical focus of the present work.

Further research into visual assessment methods has indicated varying levels of reliability. Studies by Bahannan [36] and Özat et al. [37] showcased high reliability for shade selection by vision, although Özat et al. [37] reported a lower reliability percentage (35%) compared to the visual assessment reliability in the study. This difference could be due to variations in the number of examiners and their clinical experience.

Conversely, Smith et al. [38] and Tung et al. [39] demonstrated the reliability of photographic shade selection using digital cameras under in vitro conditions on extracted teeth. These findings contrast the less favorable results for photography observed in the study.

Limitations of this study include its small sample size, which might restrict the generalizability of the results. Additionally, the study focused exclusively on maxillary right central incisors without dental anomalies, limiting the applicability of the findings to other dental conditions. The number of evaluators and the range of shade selection tools were also constrained, potentially affecting the reliability of the visual and photographic methods. Another point to consider is that in our study, instrumental methods (scanning and photography) were performed by one single operator to control the human factor; however, it is reported that although instrumental shade selection (spectrophotometer and scanning) demonstrated higher inter and intrareliability [33, 40], the operator skill and experience cannot be ignored.

It should be noted that the high reliability of the intraoral scanner may be specific to the 3Shape TRIOS system used in this study. Different scanner brands, software updates, and clinical conditions may yield varying results.

Future studies could enhance comprehensibility and outcomes by increasing the number of examiners and participants, incorporating a wider variety of scanners, cameras, and smartphones, and including spectrophotometer analysis for comparison. Advancements in technology, including the development of more sophisticated color‐matching tools and the application of artificial intelligence, promise to improve the accuracy and consistency of shade selection in clinical practice, potentially reducing reliance on visual assessment and subjective evaluation.

It is worth noting that a preprint of this article has been previously published [41].

## 5. Conclusions

The outcomes of this diagnostic examination have led to several key conclusions regarding the reliability of different shade selection methods in dentistry:

1. Significant variability in reliability: A notable disparity exists in the reliability of the various methods for shade selection. This finding underscores the importance of selecting an appropriate method based on its proven reliability to ensure accurate shade matching in dental restorations.
2. Superior reliability of intraoral scanning: The TRIOS 3shape intraoral scanner emerged as the most reliable method for shade selection among those tested. Its high reliability makes it a valuable tool for achieving precise color matching in dental restorations, enhancing esthetic outcomes and patient satisfaction. However, it should be utilized with exact control of confounding factors.
3. Visual assessment over photographic methods: Despite its subjective nature, the traditional visual assessment method proved more reliable than photographic methods for shade selection. This highlights the effectiveness of experienced clinical judgment, although it still falls short of the reliability offered by the intraoral scanner.
4. Limited reliability of photographic methods: SLR camera and smartphone photographic methods demonstrated poor reliability in shade selection. Given their lower reliability, these methods should be used cautiously for color determination in clinical settings where accuracy is paramount.

These conclusions suggest a hierarchy of reliability among the shade selection methods studied, with intraoral scanning leading the way. While visual assessment remains a viable option, especially in scenarios where advanced technology is unavailable, photographic methods should be approached with an understanding of their limitations. The findings advocate for increased adoption of intraoral scanners in clinical practice to enhance the accuracy of color matching in dental restorations.

## Author Contributions

**Faezeh Atri:** conceptualization, methodology, supervision, project administration, writing – review and editing. **Faraz Rahmani:** investigation, formal analysis, data curation, writing – original draft, resources. **Maryam Memarian:** investigation, validation, writing – review and editing, visualization. **Parsa Pirooz:** investigation, software, formal analysis, data curation, writing – original draft, visualization.

## Funding

The authors declare that there are no funding sources to report for this study.

## Ethics Statement

This research received approval from the Ethics Committee of Tehran University of Medical Sciences (ID: 9311272021).

## Conflicts of Interest

The authors declare no conflicts of interest.

## Data Availability

Data are available upon request from the authors.
